# Serotype-Dependent Effects on the Dynamics of Pneumococcal Colonization and Implications for Transmission

**DOI:** 10.1128/mbio.00158-22

**Published:** 2022-03-15

**Authors:** Annie R. Abruzzo, Surya D. Aggarwal, Molly E. Sharp, Gavyn Chern Wei Bee, Jeffrey N. Weiser

**Affiliations:** a Department of Microbiology, New York University Grossman School of Medicine, New York, New York, USA; Albert Einstein College of Medicine

**Keywords:** Streptococcus, capsule, colonization, pneumococcus, transmission

## Abstract

Capsule-switch mutants were compared to analyze how serotype affects the success of Streptococcus pneumoniae (Spn) during colonization and transmission. Strains of multiple serotypes were tested in highly susceptible infant mice, both singly and in competitive assays. Our findings demonstrated a role of serotype, apart from genetic background, in competitive success of strains, but this depended on timing postinoculation. As is the case for natural carriage, there was a hierarchy of success among serotypes using capsule-switch strains. The long-term dominance of a serotype was established within the first 4 h after acquisition, suggesting an effect independent of Spn-induced host responses. The hierarchy of serotype dominance correlated with decreased clearance rather than increased growth *in vivo*. Competitive assays staggering the timing of challenge showed that the first strain to dominate the niche sustained its competitive advantage, potentially explaining how increased density from delayed early clearance could result in serotype-dependent success. Effector molecules of intrastrain competition (fratricide), regulated by the competence operon in a quorum-sensing mechanism, were required for early niche dominance. This suggested a winner-takes-all scenario in which serotype is a major factor in achieving early niche dominance, such that once a strain reaches a threshold density it is able to exclude competitors through fratricide. Serotype was also an important determinant of transmission dynamics, although transit to a recipient host depended on effects of serotype different from its contribution to the dominance of colonization in the donor host.

## INTRODUCTION

Capsular polysaccharide (CPS) plays a dominant role in the biology of the opportunistic pathogen, Streptococcus pneumoniae (Spn, the pneumococcus), and is the basis of its serotypes (1). Although more than 100 structurally different and antigenically distinct serotypes have been described, for reasons that remain unknown only a few typically account for the majority of Spn infections. This has allowed for the deployment of CPS-based vaccines targeting only a limited number of Spn serotypes. Spn vaccines, particularly conjugate vaccines administered to young children, have reduced the burden of invasive Spn infection in the community caused by included serotypes. Much of this effect, which protects the unvaccinated population (herd immunity), has been attributed to a reduction in the transmission of strains of included serotypes (2). Immune pressure from widespread use of these vaccines, however, has led to an increase in the prevalence of infection by nonvaccine serotypes (3). This “serotype replacement” has caused a need to increase the number of types of CPS included in current vaccine formulations and points to the need to better understand the ecology of Spn, and in particular the interactions among pneumococci.

The phenomenon of serotype replacement suggests that prior to the selective pressure of immunization, nonvaccine serotypes were effectively being suppressed by the more common vaccine serotypes. In this regard, several lines of evidence indicate that Spn strains compete with one another when occupying their niche along the mucosal surfaces of the human nasopharynx (4). Although the simultaneous colonization by strains of different serotype occurs, it is far less common than would be predicted considering high rates of Spn carriage in the community (5). Spn strains express an array of quorum-sensing regulated factors capable of killing other pneumococci not expressing cognate immunity factors. The *blp* locus encodes a family of bacteriocins (pneumocins), which have been shown to promote competitive interactions between different strains of Spn (6). Additionally, a separate quorum-sensing system controlling the competence regulon causes lineage-independent killing of Spn (referred to as “fratricide”) that have not achieved the necessary population size to turn on immunity factors also regulated by this system (7). The competitive fitness of clinical isolates and isogenic “capsule switch” strains, which differ only in the serotype-determining *cps* locus, have been demonstrated in mouse models of Spn colonization. In a study by Trzciński et al., following inoculation of a mixture of strains into adult mice, there was a reproducible hierarchy of Spn serotypes independent of genetic background (8).

There is, however, minimal understanding of how competitive interactions among Spn contribute to the dominance of particular serotypes. In this report, we employed an infant mouse model of colonization to explore the ways serotype affects the dynamics of Spn colonization. Infant mice are more susceptible (1,000-fold lower colonizing dose), are colonized at a higher density, and show prolonged colonization compared to adults (9). These features recapitulate natural age-dependent characteristics of carriage in humans. Importantly, in quantitative studies of infant mouse colonization, far more pneumococci are detected in the output compared to the input, ensuring that growth effects are taken into consideration. Additionally, host-to-host transmission of Spn can be modeled in infant mice (10).

Our findings show resistance to mucosal clearance and early dominance of the mucosal niche are the key attributes for serotype-dependent success during colonization. The dominance of this niche in donors, however, is not a requirement for transmission to their recipients.

## RESULTS

### Modeling competitive interactions among pneumococci.

**FIG 1:**
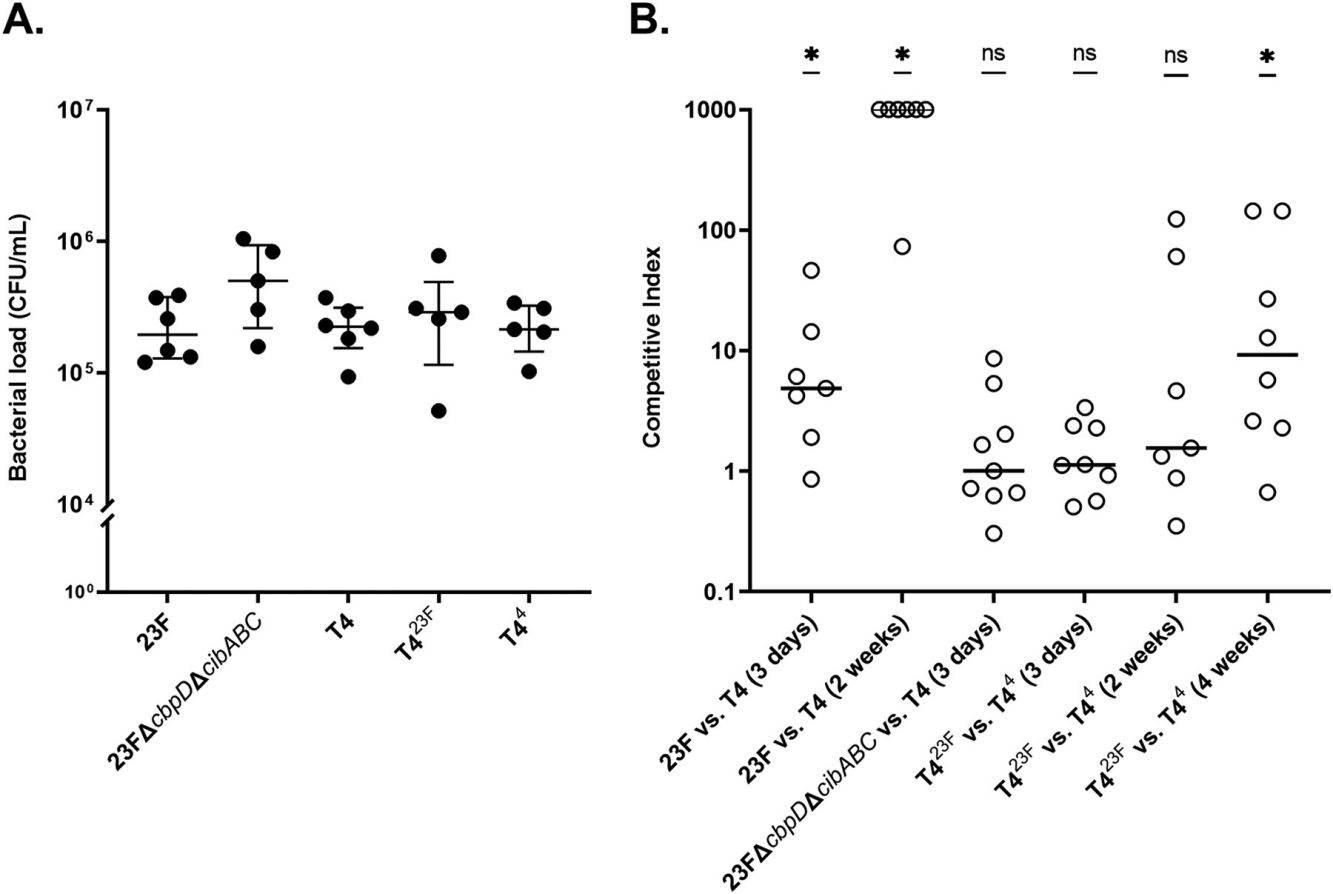
Role of Spn serotypes in facilitating competition during colonization. (A) Density of the strain of Spn indicated cultured from URT lavages at 3 days following IN inoculation of 4-day-old pups. Median values are indicated, with each symbol representing a single pup. No significant differences in colonization levels between strains were found when using a Kruskal-Wallis test, followed by Dunn’s multiple comparison test. (B) The roles of serotype, genetic background, and colonization duration on competitive interactions between strains. Pups were each challenged as described above with an even mixture of the strains indicated, and URT lavages were collected at the time point indicated to determine bacterial density with strains distinguished by colony immunoblotting using serotype-specific typing sera. A competitive index was calculated by comparing the ratio of the two strains cultured in URT lavages to the ratio in the inoculum. A competitive index greater than 1 indicates a higher proportion of the serotype 23F compared to serotype 4 strain. Competitive index values of 1,000 were used for pups where only the serotype 23F strain was detected. Median values are indicated, and each symbol represents the competitive index from a single pup. Competitive indices were compared to a hypothetical median of 1, the value representing no competition, using the Wilcoxon Signed Rank test. *, *P < *0.05; ns, not significant.

First, we demonstrated the use of infant mice to model competitive interactions between Spn using two clinical isolates of different serotypes (4 and 23F). We inoculated 4-day old mice intranasally (IN) with 10^2^ CFU/pup. Colonization density was assessed in retrograde lavages of the upper respiratory tract (URT) at 3 days postinoculation (p.i.) when peak colonization levels are observed. Differences in serotype were distinguished by colony immunoblotting. Both clinical isolates colonized similarly when given alone (Fig. 1A), but the serotype 23F isolate outcompeted the serotype 4 isolate when given together by 3 days p.i. (Fig. 1B). By 2 weeks p.i., the serotype 23F isolate almost completely dominated the niche. Next, to determine the contribution of serotype differences in competitive interactions, we tested isogenic constructs in which the serotype 4 or 23F *cps* locus was transformed into the same genetic background (T4Δ*cps*). Both constructs colonized at equivalent density when inoculated singly (Fig. 1A). In contrast to the clinical isolates, competition between the isogenic constructs T4^4^ and T4^23F^ was minimal at 3 days p.i., suggesting a contribution of factors other than serotype (Fig. 1B). At a later time point (4 weeks p.i.), however, there was a competitive advantage of the serotype 23F strain independent of genetic background. Together, these observations demonstrated that serotype itself contributes to the outcome of competition among Spn during colonization but that this effect is time dependent.

### Kinetics of serotype-dependent effects on competitive colonization.

**FIG 2:**
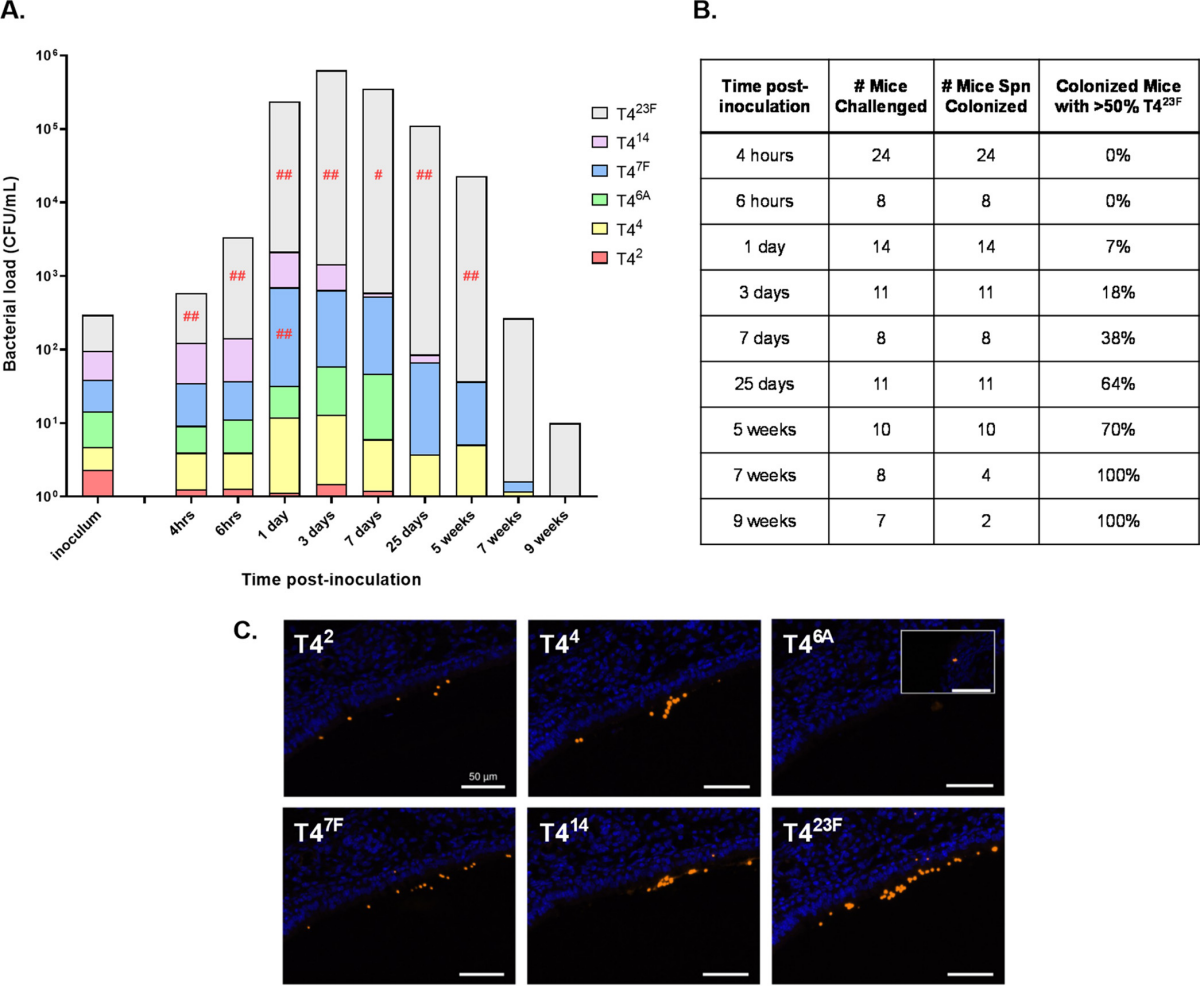
Relative abundance of serotypes in multi-strain colonization over time. Pups were challenged IN at 4 days of age with an even mixture of six capsule-switch constructs at a dose of 10^2^ CFU. (A) Bar height is the mean CFU/mL (shown in log-scale on *y* axis) recovered in URT lavages at the postinoculation time indicated. Each bar is divided (shown in linear scale) to represent the mean proportion out of 100% of each serotype as determined by colony immunoblotting. The inoculum is shown as total CFU delivered. Differences in serotype proportion between the lavage and inoculum were compared to a hypothetical median of 0 using the Wilcoxon Signed Rank test. (Relative serotype proportion in URT lavages was compared to the proportion present in the inoculum for a given experiment to account for slight variations during inoculum preparation.) Red symbols indicate a significant increase in serotype proportion during colonization relative to the inoculum. ^#^, *P < *0.05; ^##^, *P < *0.01. (B) Dominance of serotype type 23F over time during multistrain colonization. The total number of mice challenged with a 6-strain inoculum for the experiments in panel A is shown. Of the mice colonized at a given time point, the percentage of mice where T4^23F^ (serotype type 23F capsule switch construct) accounts for greater than 50% of cultured Spn in lavages is shown. (C) URT tissue sections of pups challenged IN at 6–8 days of age with an even mixture of six capsule-switch constructs at a dose of 10^2^ CFU were examined using immunofluorescence. Pups were sacrificed 24 h postinoculation and adjacent sections of fixed tissue stained for Spn (orange) with the individual serotype-specific sera indicated. Representative images are from the same site along the nasal septum in the same mouse with tissue stained with DAPI (blue). As no T4^6A^ was seen at this site, a separate image is used to document its detection (inset).

We then expanded our model to simultaneously track six isogenic strains of different serotypes (2, 4, 6A, 7F, 14, 23F) constructed in the T4Δ*cps* genetic background, spanning the full duration of carriage from 4 h to 9 weeks p.i. Following an equal, low-density inoculum of all six strains, all colonized successfully but as early as 4 h p.i. there was a significant increase in the proportion of the serotype 23F strain compared to the inoculum (gray bars, Fig. 2A). The relative success of the serotype 23F strain increased with time as the density of total colonizing Spn rose over the first 3 days p.i. and continued as the organism was mostly cleared about 9 weeks p.i. The dominance of one strain, T4^23F^, was also demonstrated by the gradual increase in the percentage of mice in which it represented >50% of the Spn cultured in lavages (Fig. 2B). This was achieved in the majority of colonized mice by 25 days and in all mice by 7 weeks p.i. The only other strain that increased relative to the inoculum was T4^7F^, but this was statistically significant only at 1 day p.i. (Fig. 2A). Both T4^2^ and T4^6A^ were essentially undetectable by 25 days p.i. These results confirmed that serotype plays an important role in competitive success during colonization.

We also questioned whether serotype-dependent competition was similar in adult mouse colonization where a much larger inoculum (10^5^ CFU dose/animal) is required to reliably establish colonization. As is the case for infant mice, at 3 days p.i. adult mice showed an increase in the proportion of the serotype 23F strain compared to the inoculum (Fig. S1 in the supplemental material). In adult mice, a dominance of T4^23F^ was observed, but this was significantly less robust compared to infants at the same time point. Lower colonization density and increased serotype diversity also typify natural adult carriage (11).

To determine whether strains were colonizing the same or distinct sites in the upper airways, we examined tissue sections at 1 day p.i. from pups given all six strains using anti-CPS sera and immunofluorescence imaging. As described previously, Spn were seen predominantly within the nasal lumen and mucosal surfaces adjacent to the nasal turbinates and proximal nasal septum. When adjacent tissue sections were stained for strains of each serotype, Spn were found in similar locations (representative images along the same site on the nasal septum shown, Fig. 2C). Consistent with the numbers cultured from lavages, more bacteria of serotype 23F were detected compared to more poorly competing serotypes 6A and 2. We concluded that isogenic Spn of different serotype were colonizing the same spatial niche, albeit at differing densities, with predominance of a single serotype emerging within hours following IN challenge.

### Serotype success during competitive colonization correlates with differences in clearance.

**FIG 3:**
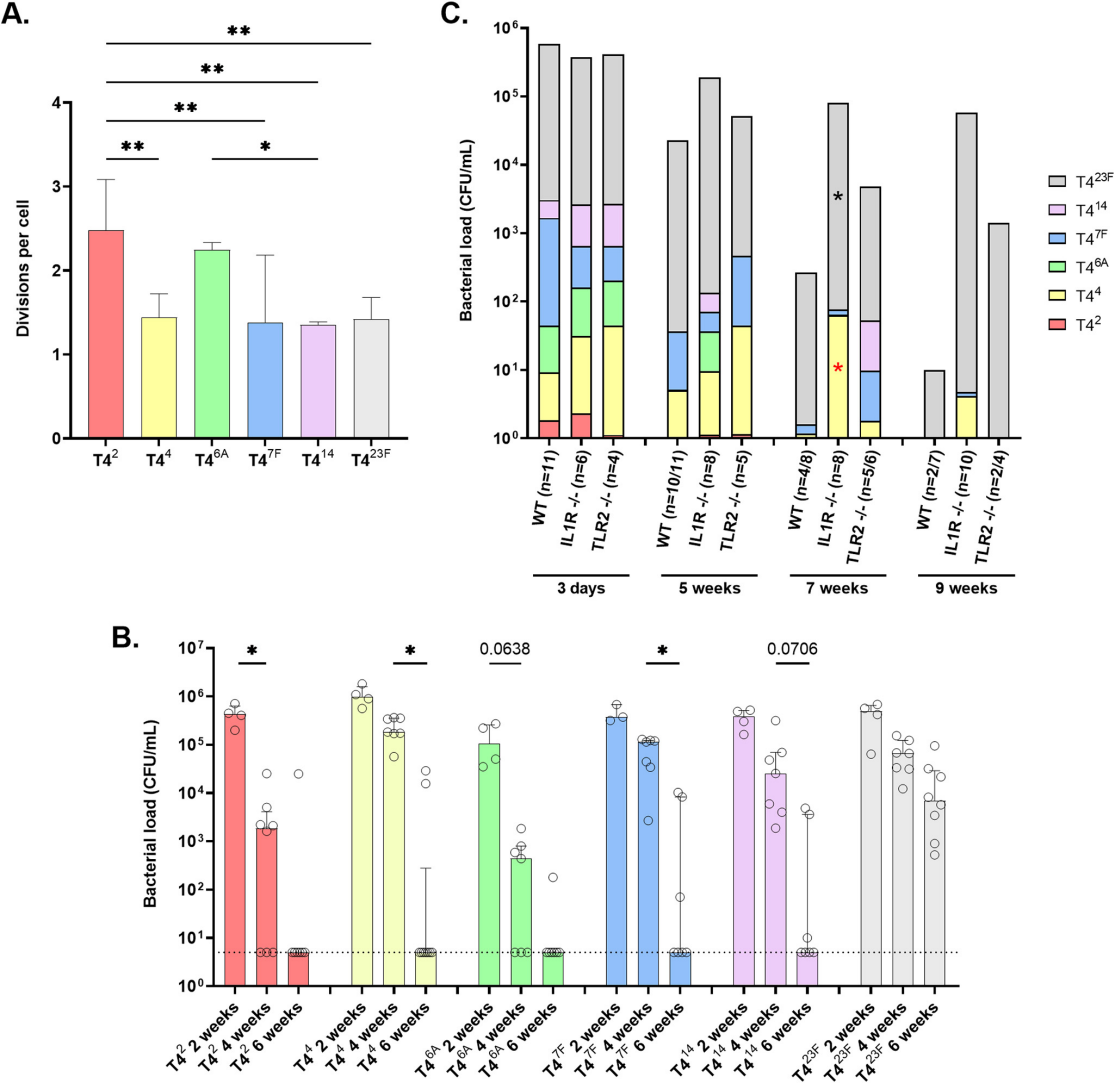
Roles of growth and clearance in serotype success. (A) Each capsule-switch construct was stained individually with carboxyfluorescein diacetate succinimidyl ester (CFSE) cell proliferation dye. Four-day-old pups were IN challenged at a dose of 10^7^ CFU with a single CFSE-stained capsule-switch construct, and URT lavages were collected at 0 and 8 h. Samples were fixed, stained with serotype specific serum, and analyzed by flow cytometry for decrease of CFSE fluorescence intensity. Data is expressed as divisions per cell with each division representing a 50% loss of the signal. Vertical bars indicate the median number of divisions per cell and interquartile range, which were compared between serotypes using an ANOVA with Tukey's multiple-comparison test. *, *P < *0.05; **, *P < *0.01. *n* = 3-4 pups/group. (B) Pups were IN challenged at 4 days of age with a single capsule-switch construct. Bacteria were cultured from URT lavages at the time point indicated with individual mice shown and bars representing median values. Differences between time points for the same construct were compared using a Kruskal-Wallis test followed by Dunn’s multiple comparison test. *, *P < *0.05. (C) The effects of innate immune factors were assessed by comparison of serotype-dependent colonization of WT, *Il1r^−/−^*, and *Tlr2^−/−^* mice. Pups were IN challenged at 4 days of age with an even mixture of six capsule-switch constructs. Spn cultured in URT lavages, at the time point indicated, were quantified and the proportion of each serotype present determined by colony immunoblotting. Numbers of mice challenged in each group are shown, and the number that remained colonized is also indicated. Differences in serotype proportion between the WT and knockout mice at the same time point were compared to a hypothetical median of 0 using the Wilcoxon Signed Rank test with red symbols (*) indicating a significant increase in serotype proportion during colonization relative to WT mice, while black symbols (*) represent a significant decrease. *, *P < *0.05.

Next, we queried whether the competitive success of T4^23F^ (and to a lesser extent T4^7F^) over strains of other serotypes was due to increased growth or decreased clearance (or a combination of both) during colonization. The *in vitro* growth of the six strains in nutrient medium was equivalent. To compare growth *in vivo*, we adapted a previously described carboxyfluorescein diacetate succinimidyl ester (CFSE) dilution assay where the fluorescent dye is used to stain bacteria prior to inoculation (12). The extent of its dilution during bacterial replication is then used as a measure of the number of cell divisions. Using this technique on Spn obtained from lavages, we were able to quantify growth over the first 8 h of colonization with each strain tested separately (Fig. 3A). Surprisingly, significantly faster growth was seen for the serotype 2 and 6A strains—the two serotypes that competed most poorly in the competitive model. Faster growth of T4^2^ and T4^6A^, however, did not result in greater colonization density at 8 h p.i. (Fig. S2), suggesting that increased replication of these strains during colonization was offset by their increased rate of clearance.

Knowing that success during competitive colonization was not associated with increased growth, we compared the persistence of each strain (tested individually) as a measure of its clearance. In this regard, we previously reported that serotype rather than genetic background is the main determinant of carriage duration—a finding also reported in clinical studies (13, 14). At 2 weeks p.i. all six strains colonized at equivalent levels (Fig. 3B). Between 2 and 4 weeks p.i., only the serotype 2 and 6A constructs showed significant clearance. By 6 weeks p.i., most mice cleared colonization except for serotype 23F. Thus, the kinetics of clearance (from fastest to slowest serotypes 2/6A> 4/7F/14 > 23F), were inversely proportional to serotype success during competitive colonization (23F> 7F> 4 > 14 > 2/6A based on 25 day data in Fig. 2A).

Host factors previously shown to affect persistence during murine colonization include innate (IL-1 and TLR-2 signaling) and adaptive immunity (T_H_17 responses) (13, 15–17). Because of the rapid selection of serotypes as early as 4 h p.i., we focused on innate immune clearance mechanisms. Spn showed more prolonged carriage in both *Il1r*^−/−^ and *Tlr*2^−/−^ mice, with most mice still colonized 9 weeks p.i. (Fig. 3C). As in wild-type (WT) mice, construct T4^23F^ became the dominant serotype, albeit with somewhat delayed kinetics, as shown by a decreased proportion of serotype 23F and increased proportion of serotype 4 at 7 weeks p.i. in *Il1r*^−/−^ compared to WT mice. We concluded that relative serotype success was associated with differences in rates of clearance and was independent of host factors known to impact carriage duration.

### Effect of timing on serotype success during competitive colonization.

**FIG 4:**
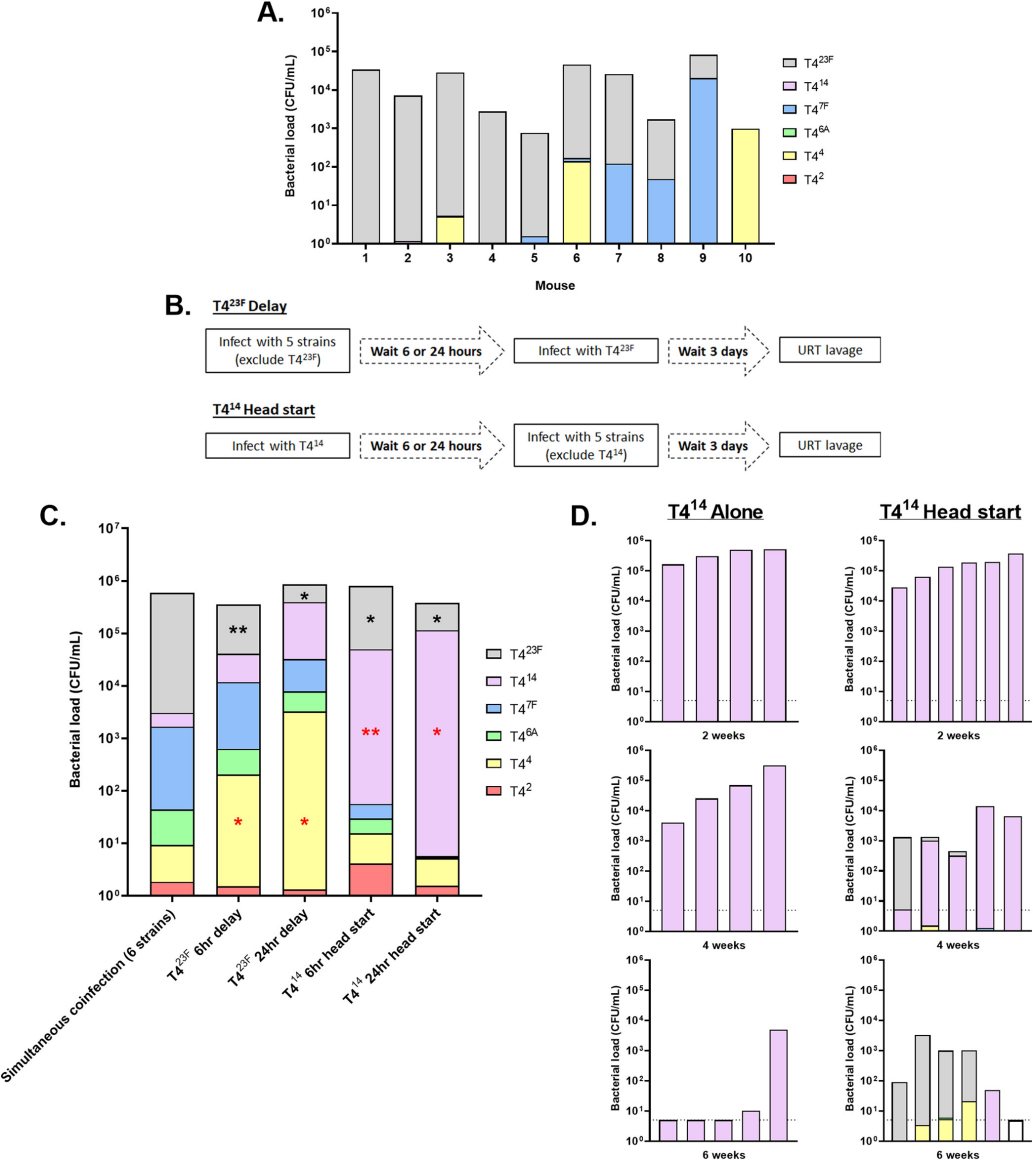
The effect of timing on serotype success during competitive colonization. (A) To show mouse-to-mouse variation in serotype presence, URT lavages were obtained 35 days postinoculation with the six capsule-switch constructs. The proportion of each serotype was determined by colony immunoblot. Data from 10 individual mice are shown. (B) Schematic of the experimental timeline. In “head start” experiments, pups were inoculated IN at 4 days of age with a single capsule-switch construct (T4^14^). Six or 24 h later, as indicated, pups were then challenged with an even mixture of the other 5 capsule-switch constructs. In “delay” experiments, pups were inoculated IN at 4 days of age with an even mixture of 5 capsule-switch constructs. Six or 24 h later, as indicated, pups were challenged with the sixth capsule-switch construct (T4^23F^). In both experiments, bacteria were recovered by URT lavages 3 days after the last inoculation and analyzed for serotype composition by colony immunoblotting. (C) The effect of a head start or delay on serotype success was determined by comparison to serotype proportions in standard 3-day colonization where all six constructs were given simultaneously. These differences in serotype proportion were compared to a hypothetical median of 0 using the Wilcoxon Signed Rank test. Red symbols (*) indicate a significant increase in serotype proportion, while black symbols (*) represent a significant decrease compared to the standard colonization. *, *P < *0.05; **, *P < *0.01. *n* = 6–10 mice/condition. (D) Pups were inoculated IN at 4 days of age with T4^14^ alone. Left-hand panels show the colonization density of T4^14^ recovered by URT lavage of individual mice at 2, 4, and 6 weeks postchallenge. Pups shown in right-hand panels were given a second inoculation with the other 5 capsule-switch constructs 2, 4, or 6 weeks after the first. Bacteria were recovered by URT lavage from these pups 3 days following the second infection, and analyzed for CFU and serotype composition by colony immunoblotting. Each bar denotes a single mouse. Dashed horizontal lines represent the limit of detection.

An interesting pattern was noted when comparing individual mice. While T4^23F^ was dominant in most mice, in others it was either a minor component or completely absent even at late time points (representative example shown at 35 days p.i., Fig. 4A). This suggested that the first occupant to gain a numerical advantage was able to dominate the niche (“winner takes all” kinetics), and this was usually but not always T4^23F^. To test this hypothesis, we modified the six-strain challenge experiment by delaying inoculation of the winner strain, T4^23F^, until 24 h after challenge with the other five serotypes and assessed colonization 3 days later (schematic in Fig. 4B). In this scenario, serotype 4 replaced 23F as the dominant strain (Fig. 4C). This same effect was seen when the delay in inoculation of T4^23F^ was as short as 6 h following the other constructs. To further test the “winner takes all” hypothesis, we gave an advantage to a construct that was consistently a “loser” by inoculating it prior to the other strains. A 24 h head start given to T4^14^ allowed it to almost completely dominate the niche. As short as a 6-h head start was sufficient for T4^14^ to become the dominant strain rather than T4^23F^. Thus, serotype success during competitive colonization depended on gaining an early advantage.

We also addressed at what point in the course of the decline in colonization density the niche becomes “open” again to new challengers. In these experiments, T4^14^ was given either a 2-, 4-, or 6-week head start before mice were challenged with the other 5 serotypes, with competitive colonization assessed 3 days later (Fig. 4D). Two weeks after T4^14^ inoculation, when all mice were still colonized at high levels (>10^5^ CFU/mL lavage), the other strains were unable to gain a foothold. Four weeks after T4^14^ inoculation, its colonization levels began to decline (10^3-5^ CFU/mL lavage), and there was limited acquisition by other strains. Six weeks after T4^14^ inoculation, when most mice had cleared T4^14^ colonization, other strains were able to establish colonization and serotype 23F Spn dominated as in naive mice. Therefore, the colonization niche becomes open to new challengers only after the occupant’s density is reduced to levels below a threshold.

### The role of quorum-sensing systems in serotype-dependent competition.

**FIG 5:**
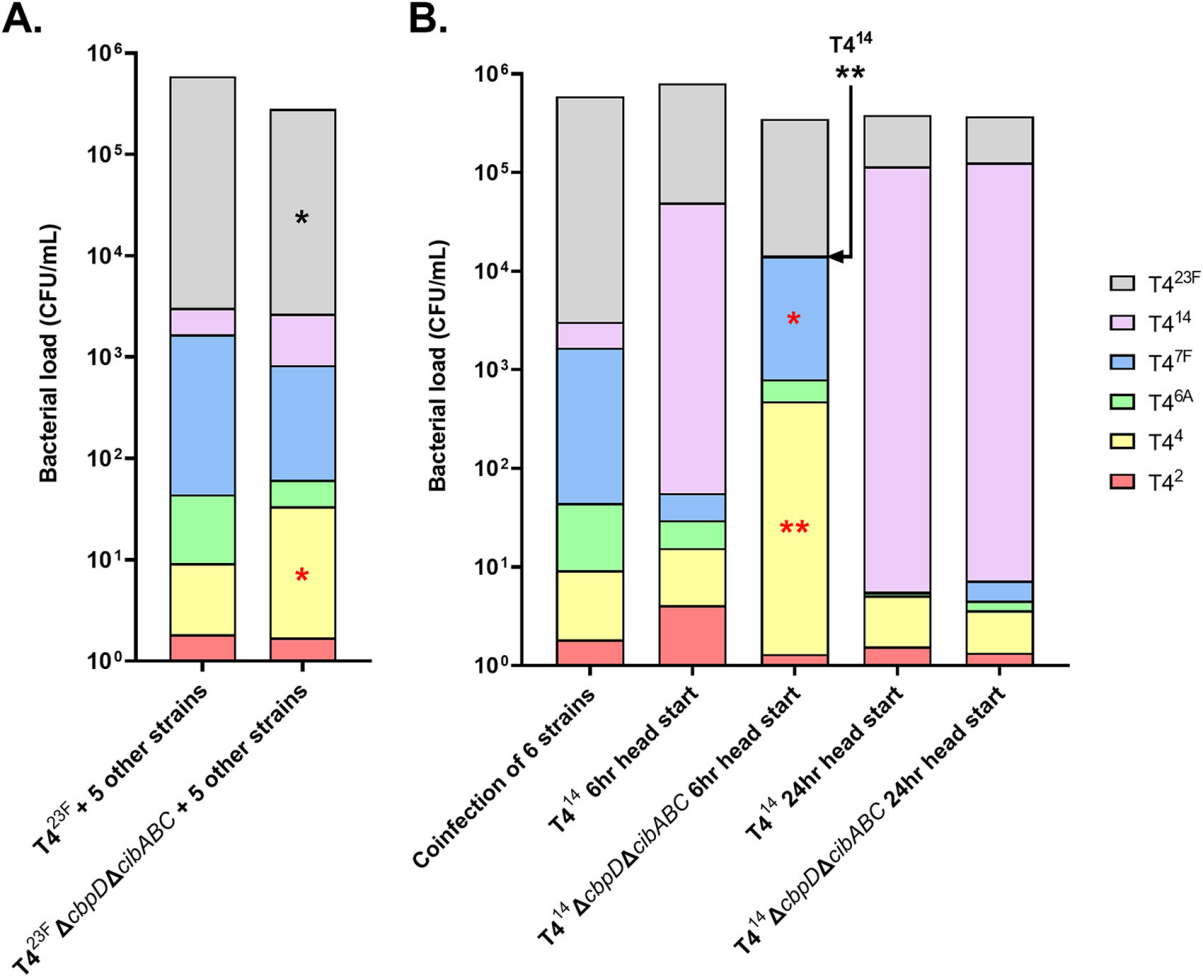
The role of quorum-sensing fratricide in serotype-dependent competition. (A) Pups at 4 days of age were IN inoculated with an even mixture of 6 capsule-switch constructs with colonization density and serotype proportion determined 3 days postinoculation. Where indicated, T4^23F^ was substituted with T4^23F^Δ*cbpD*Δ*cibABC*, which no longer expresses the fratricide effectors. *n* = 10–16 mice per condition. (B) In “head start” experiments, pups were IN inoculated with a single capsule-switch construct, either the T4^14^ or T4^14^Δ*cbpD*Δ*cibABC*, and 6 or 24 h later, as indicated, IN inoculated with an even mixture of the other 5 capsule-switch constructs with colonization density and serotype proportion determined 3 days after the last inoculation. *n* = 7–9 mice/condition. Differences in serotype proportion were assessed between pups infected with the parent strain and corresponding Δ*cbpD*Δ*cibABC* construct, with red symbols (*) indicating a significant increase while black symbols (*) represent a significant decrease. Differences in serotype proportion were compared to a hypothetical median of 0 using the Wilcoxon Signed Rank test. *, *P < *0.05; **, *P < *0.01.

Our observations confirmed that strains must compete for a niche in the URT that is either resource and/or spatially limited, since maximal colonization density was ∼10^6^ CFU/mL lavage regardless of the number of strains inoculated. Furthermore, the colonization density of poorly competing strains was lower when co-challenged with five other serotypes compared to single inoculation (such as T4^14^ at 7 and 25 days in Fig. 2A compared to Fig. 4D). Since the data indicated that a serotype-dependent competitive advantage correlated with rapid attainment of a higher colonization density, we examined the role of Spn quorum-sensing systems. A mutant unable to express the quorum sensing peptide BlpC, which activates the *blp* bacteriocins operon, was constructed in T4^23F^, and this construct was substituted for its parent in the competitive colonization assay with the other 5 strains. T4^23F^Δ*blpC* was still able to dominate the niche 3 days later (data not shown). In contrast, knocking out both fratricide effectors CbpD and CibAB resulted in a small but significant decreased prevalence compared to the parental strain, T4^23F^ (Fig. 5A). With the dominance of T4^23F^ impaired by the loss of fratricide effectors, the proportion of T4^4^ was increased significantly. To further examine the role of fratricide, a T4^14^Δ*cbpD*Δ*cibABC* construct was substituted for T4^14^ in a “head start” experiment. When inoculated 6 h prior to the other five strains, T4^14^Δ*cbpD*Δ*cibABC* was no longer able to dominate the niche (Fig. 5B). In contrast, T4^14^Δ*cbpD*Δ*cibABC* was still able to dominate the niche when given a head start of 24 h. Together, these results indicate that fratricide contributes to early niche dominance. However, once a strain has fully filled its niche, which occurs about 24 h p.i. (18), fratricide is no longer necessary to inhibit competitors.

We also examined whether fratricide contributed to competition between nonisogenic clinical isolates. Without the effectors CbpD and CibAB, the serotype 23F isolate no longer showed a competitive advantage over the serotype 4 isolate, and both colonized at equivalent levels when tested singly (Fig. 1A and B).

### The impact of serotype-dependent competition on transmission.

**FIG 6:**
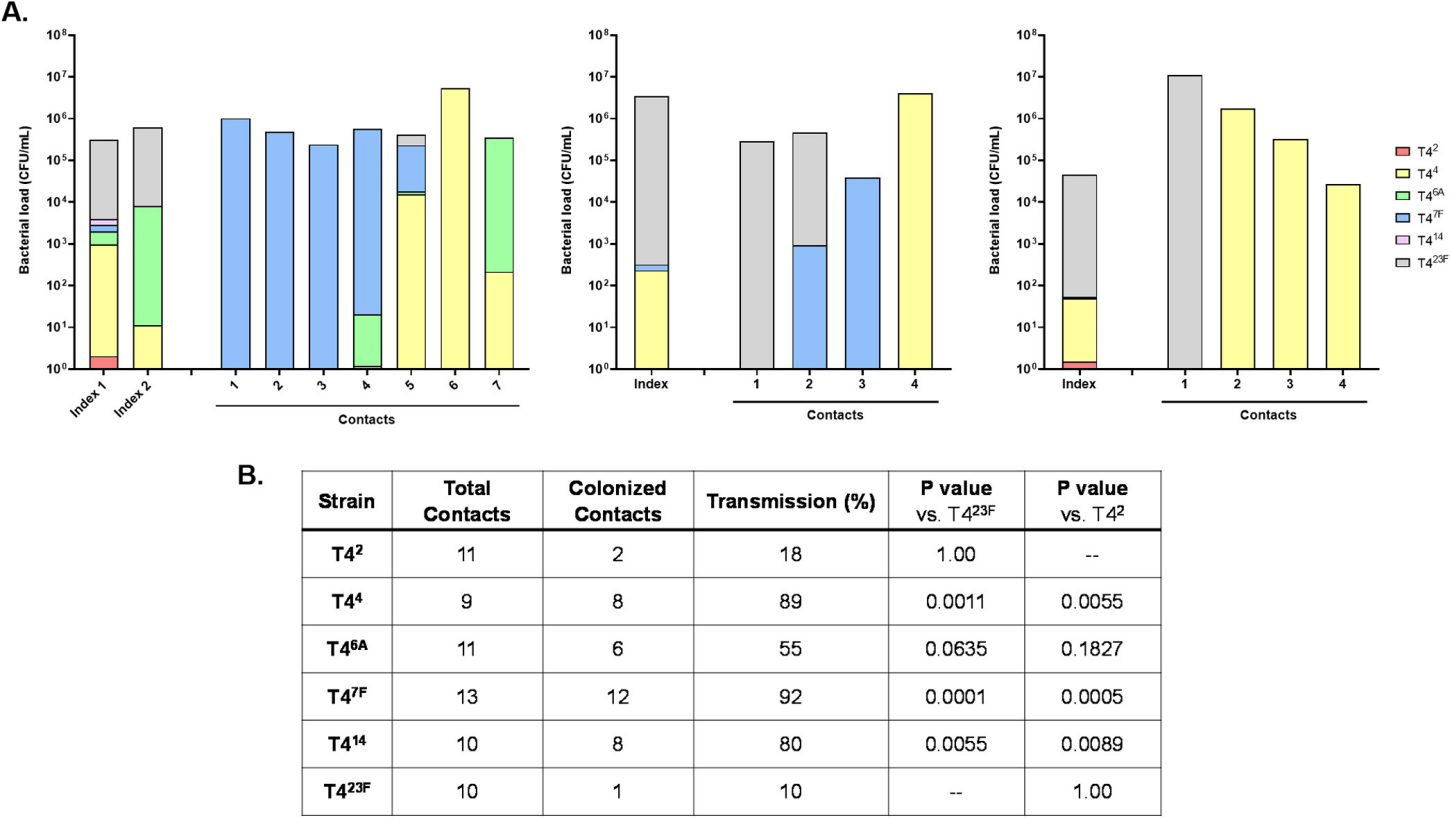
The impact of serotype-dependent competition on transmission. Within a litter, one in three to four pups (index mice) were challenged IN at 4–7 days of age with (A) an even mixture of six capsule-switch constructs or (B) a single capsule-switch construct. Four days later, all pups in the litter were inoculated IN with influenza A virus. Transmission to contact pups was assessed in URT lavages 5–6 days after virus inoculation. For experiments using the mixed strain inoculum, the proportion of each serotype in index and contact mice at the time of sacrifice was determined by colony immunoblotting, with individual colonized mice from three litters shown in A. Each bar denotes a single mouse. For pups challenged with single capsule-switch constructs, the transmission rate is shown with differences compared to T4^23F^ or T4^2^ strains using the Fisher’s Exact Test.

One of the expected consequences of niche dominance might be an increased opportunity for transmission to new hosts. This was modeled by inoculating 1 in every 3 to 4 infant mice (the index mice) within a litter with constructs of all six serotypes. This was followed 4 days later with coinfection of all infants in the litter with influenza A, which increases secretions, bacterial shedding, and the rates of transmission to the littermates (contacts) (19). When the mice were sacrificed to analyze colonization (5 days p.i. with virus), the index mice coinfected with influenza showed a similar pattern of serotype dominance compared to non-influenza infected mice with a dominance of T4^23F^ in about half (Fig. 6A, showing three representative litters). Surprisingly, the pattern in the contact pups was very different. Transmission of T4^23F^ was dominant in only 2/15 colonized contact pups. T4^7F^, which was detected in small numbers in only 2/4 index mice, was the most commonly transmitted strain (7/15 colonized contacts) and was the dominant strain in 6/15 colonized contact pups. Strain T4^4^ was also commonly transmitted (7/15 colonized contacts). We then compared the ability of each of the constructs to transmit when given singly to index mice in this model (Fig. 6B). Rates of transmission were significantly lower for T4^23F^ and T4^2^ compared to strains of other serotypes, with serotypes 7F and 4 being transmitted most efficiently. Thus, a strain’s chances of establishing colonization in a new host was more dependent on serotype-related differences in transmission rate than its dominance in the original host.

## DISCUSSION

While there has been much attention paid to the ability of encapsulated Spn to cause invasive infections, these disease outcomes represent biological dead ends for the organism (1). This report examined the role of Spn’s CPS in the two critical steps in the life cycle of the bacterium, colonization of the URT and host-to-host transmission from that site. There were several important aspects of our approach. Our models relied on capsule-switch constructs enabling a focus on serotype-dependent effects separate from any contributions of genetic background. Our findings reiterate the existence of a hierarchy of serotypes in successful colonization (8). Comparing competitive interactions among serotypes in a highly tractable infant mouse model allowed for a number of new insights into the impact of serotype on colonization and transmission. Firstly, our data using clinical isolates demonstrated marked interstrain competition among pneumococci, but also showed that serotype is not the only bacterial factor involved in these interactions. Serotype-mediated competitive interactions were dependent on the duration of colonization, in some instances occurring rapidly and others requiring weeks to manifest. For example, competition was noted between clinical isolates (serotypes 4 and 23F) by 3 days p.i., but in an isogenic background the effect of these serotypes was not detected until 4 weeks p.i. Secondly, a striking observation of the six-serotype model was that long-term competitive success during colonization was predicted by very early interactions among strains. The “winner” (serotype 23F), which consistently prevailed over a 9-week period of colonization, showed a significant advantage over other serotypes as early as 4 h p.i. Further data from experiments varying the timing of inoculation showed that a delay or head start of as little as 6 h was sufficient to turn a “winner into a loser” or a “loser into a winner,” respectively. Together, these results showed the critical advantage for early niche dominance when Spn strains compete. Lastly, we demonstrated an important role of conserved, quorum-sensing responses in Spn (fratricide effectors regulated by the competence program) in early niche dominance leading to competitive success. However, dominance of the URT niche was not sufficient for greater serotype success in transmission, suggesting that other bacterial factors affect this step in the Spn life cycle.

So how can strains that are otherwise isogenic display such marked competition? The importance of early niche dominance and fratricide effectors suggest a plausible mechanism. The strain that first reaches a threshold density senses its population size by the concentration of the competence-stimulating pheromone CSP, which then signals the upregulated expression of immunity factors ComM and CibC, among other competence-regulated genes (7). These immunity factors enable survival when the effectors of fratricide, the lysin CbpD and bacteriocins CibAB, are expressed in a later phase of the competence program. These effectors require cell–cell contact, and our analysis of tissue sections shows serotypes co-localized along the mucosal surfaces (20). Thus, early niche dominance is facilitated by achieving the threshold quorum density first and using that advantage to eliminate nearby competitors. Once the URT is occupied at its capacity, which we previously showed requires about 24 h, it appears that fratricide no longer provides a benefit and competitors are effectively excluded, likely by resource or spatial limitations (18). This model suggests that there is strong selective pressure to attain the threshold quorum quickly following acquisition, and that serotype is a factor in these events.

We considered whether differences in serotype affect early growth, clearance, or a combination of both during colonization. By 4 h p.i., when serotype-dependent competition is first detectable, minimal growth has taken place (on average <2 divisions/cell) and serotype-associated differences during *in vivo* growth did not correlate with competitive success. In fact, serotypes that fared most poorly (2, 6A) displayed faster growth without achieving a higher density, suggesting these have a higher turnover rate. It is possible their faster growth leads to greater exposure of their cell surfaces, making them more susceptible to fratricide effectors targeting the cell wall and membrane. We focused, therefore, on serotype-specific differences in clearance. The very short time frame (<4 h) made it unlikely that host responses were critical determinants of serotype-dependent success. A more likely explanation is that after arriving on the mucosal surface of the URT, certain serotypes are retained more efficiently. This retention capacity, furthermore, correlated with duration of colonization or persistence when strains of different serotypes were tested individually.

The different physical properties of CPSs could affect adherence of the bacterium to host tissues or the mucus layer and, thereby, inhibit their mechanical clearance. In adherence assays with epithelial cells in culture, however, the presence of capsule is inhibitory as it shields underlying bacterial adhesins (21). We previously described that in the first hours after acquisition, Spn is found associated with the loose mucus layer and that in the absence of capsule Spn is more quickly cleared by mucociliary flow (22). At later time points during stable colonization, Spn localizes to the firm mucus layer overlying the epithelial surface (the glycocalyx). Shielding of underlying surface structures with a sticky, hydrophobic substance might allow for retention within the firm mucus layer. Prior comparison of antibody binding to surface protein adhesins in capsule-switch constructs showed increased shielding (less exposure of underlying antigens) by the serotype 23F strain compared to strains of several other serotypes (23). Thus, effective shielding of the bacterial surface might be more important than exposure of putative adhesins in bacterial retention during early events of colonization. We speculate, therefore, that the physical characteristics of some CPSs enhance adhesive interactions inhibiting clearance.

This “intrinsic property” of certain CPSs could explain why limited arrays of serotypes are more prevalent across surveys of natural carriage (24). In this study carried out in mice, it was usually but not always the strain with 23F CPS that dominated among the six serotypes tested. Serotype 23F, which is targeted by current Spn vaccines, was among the most common of colonizing serotypes in the prevaccine era, although the hierarchy of serotypes in murine studies might not parallel that in the natural host (24). Our findings have implications for serotype replacement due to vaccine immune pressure. If success is due to the intrinsic physical properties of certain CPSs, many rare serotypes may lack the capacity to compete successfully and, as a consequence, the potential for serotype replacement might be limited in scope. A further implication is that in the prevaccine era the accumulation of immunity in the population to serotypes circulating widely would be expected eventually to select against the ascendancy of only a few serotypes. Therefore, the effect of serotype on competitive rather than immune selective pressure may be responsible for the limited serotype diversity seen in Spn carriage.

A number of attributes of CPSs have previously been proposed as correlates of serotype-dependent success during carriage. These include the main mechanism of clearance during Spn disease via opsonization by complement, with or without antibody, and subsequent clearance by professional phagocytes. The critical period for early success noted in our study, however, makes it unlikely that there would be a sufficient interval to allow for an influx of phagocytes. Additionally, studies with complement-deficient or -depleted mice show that it plays little role in colonization dynamics and naive infant mice contain no anti-Spn antibodies (15, 25). Other CPS attributes linked to patterns of serotype prevalence are surface charge (measured experimentally as zeta potential) and complexity of the CPS (compared as the number of carbons per repeat unit) (26, 27). Our study included two serotypes of neutral charge (7F, 14) and serotypes with similar complexity (14, 23F), which demonstrated very different degrees of success in competition studies. There was also no correlation of serotype success with capsule thickness, as previously measured for the capsule-switch constructs using a microscopic quantification with a FITC-dextran exclusion assay (28). It has also been proposed that serotype prevalence during carriage correlates with the length of the lag phase during growth in nutrient medium (29). However, in our study using isogenic constructs there was no detectable difference in growth characteristics under these growth conditions. We did observe serotype-related differences in growth under nutrient-limiting conditions, such as in tissue culture medium, and in survival without nutrients (in PBS). Because of these observations, our study avoided the potentially confounding effects of *in vitro* comparisons, including in adherence assays. Increased chain length has previously been linked to the efficiency of quorum sensing *in vitro* and to adherence in the nasopharynx (30, 31). There were, however, no consistent differences in clumping or chain formation observed during colonization in nasal tissue sections.

As previously noted, simultaneous carriage of Spn isolates is not uncommon, raising the question of how strains coexist (32). We demonstrated that once the URT niche becomes occupied with one strain, incoming challengers are unlikely to gain a foothold. After colonization of the resident strain falls below a threshold density, however, the niche is opened to new challengers, including those of different serotype. We also observed that co-colonization occurs when two or more strains arrive in the same host at the same time. This would not be a rare event considering the high rates of Spn colonization and frequency of transmission events, particularly among young children.

We expected that the dominant serotype in the nasopharynx of index mice would have a strong advantage during transmission. This was not observed. Rather, serotypes present at low density in the index mice were able to successfully transit to new hosts. The serotype 23F strain, which became dominant during colonization, showed a relative defect compared to other serotypes in transmission when tested singly and was found in only a minority of contact pups during competitive transmission experiments. It is possible that the adhesive characteristics that allow this serotype to be retained so effectively in the URT niche make it less likely to be released for transit to a new host. We previously showed that T4^23F^ (and T4^2^), which transmits relatively poorly, is shed from infant mice in lower numbers compared to the other capsule-switch constructs (28). Finally, there are common features of competitive success during colonization and transmission. Once a strain is acquired via transmission and becomes established in the URT, new incoming challengers are blocked in a mechanism also dependent on fratricide (an owner-intruder competitive strategy) (18).

In conclusion, our findings demonstrate that strain dynamics during both colonization and transmission need to be taken into consideration to account for serotype prevalence.

## MATERIALS AND METHODS

### Bacterial culture.

Unless otherwise specified, pneumococcal strains (described in Table S1) were grown statically in tryptic soy (TS) broth (Becton, Dickinson) at 37°C. Upon reaching the desired optical density of 1.0 at 620 nm, cells were washed and diluted in sterile phosphate-buffered saline (PBS) for inoculation. The diluted strains were mixed together at the desired density to generate inocula containing equal proportions of strains. For quantitative culture, serial dilutions were plated on TS broth–streptomycin (200 μg/mL) agar supplemented with either 5% sheep blood or catalase (6,300 U/plate; Worthington Biochemical Corporation) and incubated overnight at 37°C with 5% CO_2_. To compare growth in nutrient conditions, capsule-switch mutants were grown in Brain Heart Infusion medium containing fetal bovine serum as previously described (29).

### Bacterial strain construction.

Capsule switch constructs in the genetic background of a streptomycin-resistant derivative of TIGR4 (T4) were constructed by transforming the unencapsulated mutant T4Δ*cps* and were previously described (28). Primers used in strain construction are listed in Table S2. Transformations were carried out using a modified version of a previously described procedure (33). Bacterial colonies were inoculated and grown in acidic Columbia broth until an OD_595_ of 0.05 followed by the addition of 5 μg/mL of 1:1 mix of CSP1 and CSP2 and 500 ng of transforming DNA. Cultures were incubated at 37°C and 5% CO_2_ without shaking for 2 h followed by plating on the appropriate antibiotic: kanamycin (250 μg/mL) or streptomycin (200 μg/mL). The mutant strains were constructed using site-directed homologous recombination to replace genes of interest. The Δ*cbpDΔcibABC* mutants were constructed in a three-step process. First, *cbpD* was replaced with the Janus cassette (via a PCR product obtained from P2500 [18]) containing an additional ∼1kb each in flanking regions upstream and downstream) in T4^23F^ (P2439) and T4^14^ (P2488) to obtain strains P2647 and P2648, respectively, by selection for kanamycin resistance. Thereafter, an unmarked, in-frame deletion of *cbpD* was made by replacement of the Janus cassette as previously described (18, 34). Strains P2647 and P2648 were transformed with a PCR fragment from P2576 (containing ∼1kb regions upstream and downstream of *cbpD* joined together (18) to obtain strains P2649 and P2651, respectively. The transformants were streptomycin resistant but kanamycin sensitive. In the final step, *cibABC* was replaced with a Janus cassette (via a PCR product obtained from P2576) in strains P2649 and P2651. The resultant Δ*cbpDΔcibABC* strains P2653 (T4^23F^) and P2654 (T4^14^) were kanamycin resistant. The Δ*blpC* strain was constructed by replacing *blpC* with Janus cassette (via a PCR product obtained from P2644 containing ∼1kb each in flanking regions both upstream and downstream of the gene) in T4^23F^ (P2439). The resultant strain P2678 was kanamycin resistant. Mutants were confirmed by PCR after each step.

### Animal studies.

All animal experiments followed the guidelines summarized by the National Science Foundation Animal Welfare Act (AWA) and the Public Health Service Policy on the Humane Care and Use of Laboratory Animals. The Institutional Animal Care and Use Committee (IACUC) at New York University School of Medicine oversees the welfare, well-being, proper care, and use of all animals, and they have approved the protocol used in this study (IA16-00538).

C57BL/6J WT and congenic *Tlr2^−/−^* and *Il1r^−/−^* knockout mice were purchased from The Jackson Laboratory (Bar Harbor, ME), and were bred and housed in a conventional animal facility. Pups were maintained with their dam until weaning at age 3 weeks. Weaned mice were fed *ad lib* the PicoLab Rodent Diet 20, a 20% protein diet formulation, and were given acidified water for consumption. Additionally, the animals were kept on a light-cycle of 12 h on, 12 h off with a temperature in the animal facility of 70°F (±2°F). Throughout the experiments, mice remained healthy and did not lose weight compared to uninfected controls.

### Colonization model.

Four-day-old pups of both sexes were given an intranasal inoculation without anesthesia, containing 10^2^ CFU of Spn suspended in 3 μl of PBS, as described previously (10). To measure colonization density, pups were euthanized at the indicated time point by CO_2_ asphyxiation followed by cardiac puncture. The URT was lavaged with 200 μl of sterile PBS from a needle inserted into the trachea, and fluid was collected from the nares. Nasal lavages were spread at various dilutions on a TS agar plate with catalase supplemented with streptomycin (200 μg/mL) to prevent the growth of contaminants. Where specified, adult mice, age 6 weeks, were challenged with 10^5^ CFU in 3 μl in PBS.

### Transmission model.

The pneumococcal transmission model with IAV coinfection was described in previous studies (10). Briefly, one in three to four pups in the litter was randomly selected and, at an age of 4 to 7 days, infected (index mice) and then returned to cage containing the dam and the other uninfected pups (contact mice). Four days later, all the pups in the litter were inoculated intranasally with 3 μl IAV/HKx31 containing 250 plaque-forming units. Five days after infection with IAV, all pups were euthanized and nasal lavages were cultured to detect bacterial transmission from the index to contact pups.

### Colony immunoblotting.

A colony immunoblot using nasal lavages obtained from Spn-colonized mice provided a quantitative assessment of relative serotype abundance. Colonies from lavages grown on TS catalase plates plus streptomycin were lifted using a 0.45-μm nitrocellulose membranes (Cytiva Protran 0.45 μm, 82 mm diameter circle, cat. # 10401116). Typical dilutions yielding a minimum of 200 colonies when plated were chosen. In order to detect the relative abundance of six serotypes present in a single sample, the nitrocellulose membrane was cut into six equal pieces, each stained with a different antibody. Air-dried membranes were incubated in 3% BSA-PBS at 4°C overnight with shaking. The membrane was incubated for 1h at RT in serotype-specific antisera (Statens Serum Institut) diluted in PBS. Antibodies to serotypes 2, 6, and 23 were diluted 1:5000; serotype 4 and 14 antibodies were diluted 1:25,000; and serotype 7 antibody was diluted 1:30,000. Blots were washed 5 times (5 min each) with 0.05% Tween 20-PBS, and incubated for 45 min at RT with alkaline phosphatase-conjugated antibody to rabbit IgG (Millipore Sigma, cat. # A3687) diluted 1:5,000. All antibody incubations were carried out in 0.01% Tween 20/0.1% BSA-PBS. The membranes were washed 5 times (10 min each) with 0.05% Tween 20-PBS and developed with 1-Step NBT/BCIP Substrate Solution (Thermo Scientific, cat. # 34042).

### CFSE growth assay.

Using a modified protocol (12), bacteria were resuspended in 1 mL PBS containing 1% catalase and 10 μM carboxyfluorescein diacetate succinimidyl ester (CFSE, ThermoFisher Scientific). Reactions were carried out at 37°C for 25 min, then bacteria were washed 3 times in PBS to remove unabsorbed CFSE. Mice were inoculated IN with 10^7^ CFU of CFSE-labeled pneumococci in 3 μL. At the time of inoculation (0 h p.i.) and after 8 h p.i., mice were sacrificed and nasal lavages were obtained and analyzed using flow cytometry. Samples were fixed, stained with typing serum specific to the capsule-type used (Statens Serum Institut) at 1:1000 dilution followed by phycoerythrin (PE)-labeled secondary antibody to rabbit IgG at 1:100 dilution, and then analyzed by flow cytometry using the LSRII flow cytometer (BD Biosciences) and analyzed using FlowJo software (Tree Star). Viability of Spn is unaffected by the CFSE stain (35). Because the CFSE dye is split evenly between two daughter cells, each 50% decline in median fluorescence intensity of CFSE in the PE-positive population (Spn) at 8 h p.i. compared to 0 h p.i. was considered a cell division.

### Immunofluorescence microscopy.

Pups infected with the combination of six capsule-switch constructs as described above (except at a total dose of 10^5^ CFU) were euthanized as per standard protocol 24 h p.i. The skin was removed from decapitated heads, which were then briefly submerged in PBS followed by fixing in 4% paraformaldehyde for 48 h at 4°C without shaking. Heads were decalcified by fully submerging heads into 0.12M EDTA solution at 4°C with gentle shaking for 7 days followed by washing in PBS 3× for 20 min. Intact heads were then processed through graded ethanol to xylene washes and infiltrated with paraffin in a Leica Peloris automated tissue processor. Five micron paraffin-embedded sections were stained with antisera probes on a Leica BondRX automated stainer, according to the manufacturer’s instructions. In brief, sections were incubated for 1 h with serotype-specific antisera (1:2,000 dilution) and then 1 h with goat anti-rabbit IgG conjugated to Alexa Fluor 594 (1:100, ThermoFisher A21207). Slides were counterstained with DAPI (5′, RT) and scanned on an Akoya Polaris Vectra imaging system. The multispectral images were unmixed and autofluorescence signal removed with the Akoya InForm software prior to exporting as TIF files.

### Statistical analysis.

All statistical analyses were performed using GraphPad Prism 9.2.0 (GraphPad Software, Inc., San Diego, CA).

10.1128/mbio.00158-22.1 FIG S1 Relative serotype abundance in multistrain colonization in adult mice. Adult mice were challenged IN at 6 weeks of age with an even mixture of six capsule-switch constructs (inoculum), at a dose of 10^5^ CFU. Bacteria were recovered at 3 days postinoculation, and the proportion of each serotype present in URT lavages determined by colony immunoblotting. Bars represent the mean CFU/mL measured and are divided to indicate the proportion of each serotype, with 14 individual adult mice and average values from these mice shown. Relative serotype proportion in URT lavages was compared to the proportion present both in the inoculum and in infant URT lavages at the same time p.i., and any differences in serotype proportion were compared to a hypothetical median of 0 using the Wilcoxon Signed Rank test. Red symbols (#) indicate a significant increase in serotype proportion during colonization relative to the inoculum. Black symbols (*) indicate a significant decrease in serotype proportion in adults relative to infants. *, #, *P < *0.05; ##, *P < *0.01. Download FIG S1, PDF file, 0.01 MB. Copyright © 2022 Abruzzo et al. 2022 Abruzzo et al. https://creativecommons.org/licenses/by/4.0/ This content is distributed under the terms of the Creative Commons Attribution 4.0 International license.

10.1128/mbio.00158-22.2 FIG S2 Each capsule-switch construct was stained individually with carboxyfluorescein diacetate succinimidyl ester (CFSE) cell proliferation dye. Four-day-old pups were IN challenged at a dose of 10^7^ CFU with a single CFSE-stained construct, and URT lavages were collected both immediately (T0, solid symbols) and after 8 h (T8, open symbols) to quantify colonizing Spn. Median values are indicated, with each symbol representing the CFU/mL measured from a single pup. Download FIG S2, PDF file, 0.01 MB. Copyright © 2022 Abruzzo et al. 2022 Abruzzo et al. https://creativecommons.org/licenses/by/4.0/ This content is distributed under the terms of the Creative Commons Attribution 4.0 International license.

10.1128/mbio.00158-22.3 TABLE S1 Bacterial strains used in this experimental work Table S1, DOCX file, 0.02 MB. Copyright © 2022 Abruzzo et al. 2022 Abruzzo et al. https://creativecommons.org/licenses/by/4.0/ This content is distributed under the terms of the Creative Commons Attribution 4.0 International license.

10.1128/mbio.00158-22.4 TABLE S2 Primers used in this study. Download Table S2, DOCX file, 0.01 MB. Copyright © 2022 Abruzzo et al. 2022 Abruzzo et al. https://creativecommons.org/licenses/by/4.0/ This content is distributed under the terms of the Creative Commons Attribution 4.0 International license.
