# Supplementary figures and images for: Serotype-Dependent Effects on the Dynamics of Pneumococcal Colonization and Implications for Transmission
Source: mBio. 2022 Mar 15;13(2):e00158-22. doi: 10.1128/mbio.00158-22 (PMC9040870; doi:10.1128/mbio.00158-22)

# S1.

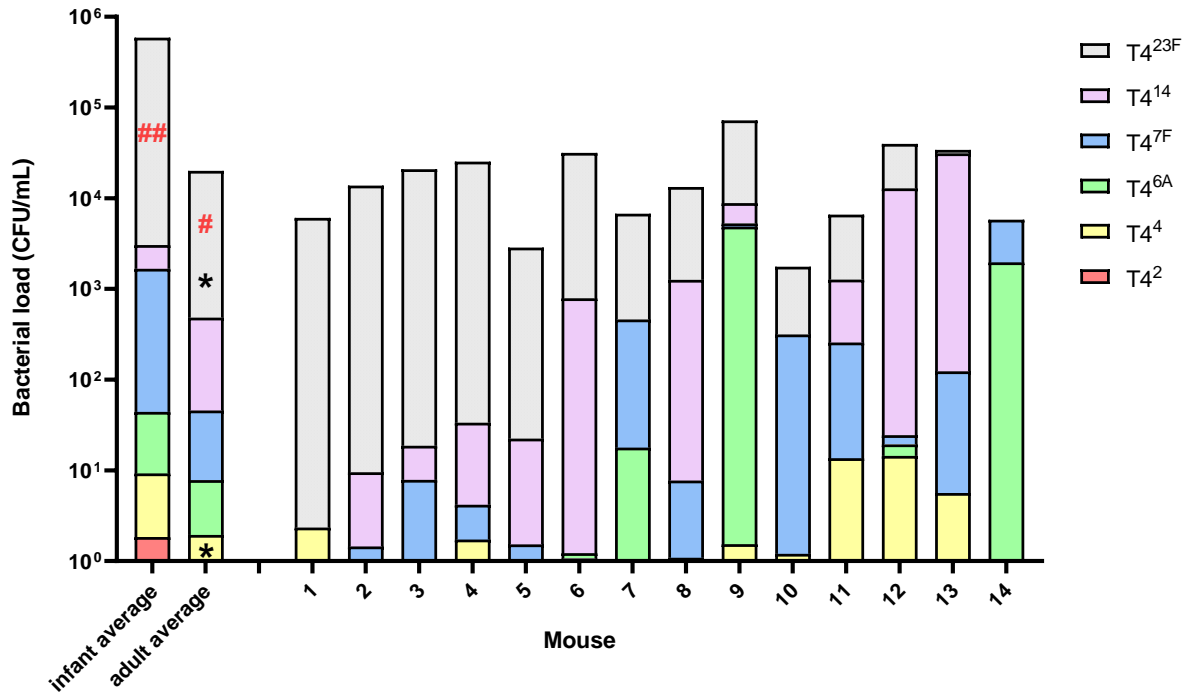

Supplement: FIG S1 [file mbio.00158-22-sf001.pdf]

# S2.

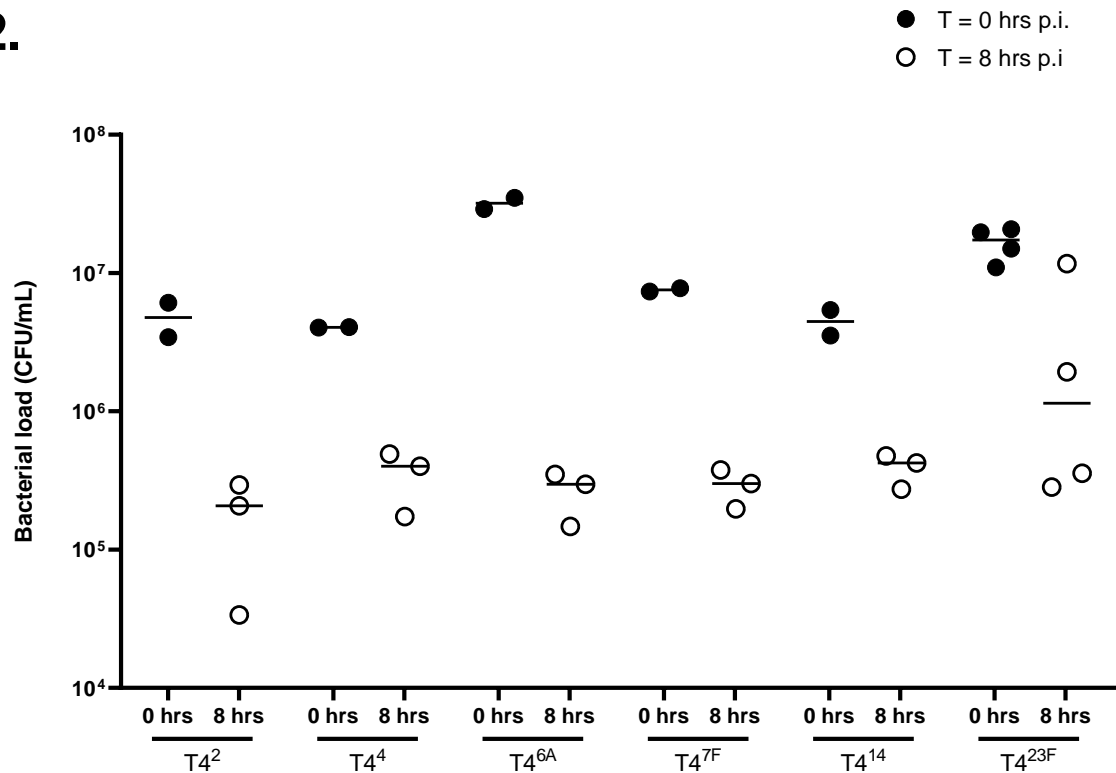

Supplement: FIG S2 [file mbio.00158-22-sf002.pdf]
