# Supplementary material for: Serotype-Dependent Effects on the Dynamics of Pneumococcal Colonization and Implications for Transmission
Source: mBio. 2022 Mar 15;13(2):e00158-22. doi: 10.1128/mbio.00158-22 (PMC9040870; doi:10.1128/mbio.00158-22)
Supplement: TABLE S1 [file mbio.00158-22-st001.docx]

**Table S1: Bacterial strains used in this experimental work**

| **Strain** | **Strain number** | **Description** | **Reference** |
| --- | --- | --- | --- |
| T4 | P2406 | Streptomycin resistant derivative of TIGR4 (T4 WT) | (1) |
| 23F | P2499 | Streptomycin resistant derivative to 23F Clinical Isolate | (2) |
| 23F Δ*cbpD*Δ*cibABC* | P2576 | P2499 with clean deletion of *cbpD* and Janus cassette replacing *cibABC* operon, kanamycin resistant | (2) |
| T4 Δ*cps* | P2422 | P2406 with *cps* clean deletion, streptomycin resistant | (3) |
| T4^2^ | P2482 | P2422 expressing type 2 capsule, streptomycin resistant | (3) |
| T4^4^ | P2438 | P2422 expressing type 4 capsule, streptomycin resistant | (3) |
| T4^6A^ | P2453 | P2422 expressing type 6A capsule, streptomycin resistant | (3) |
| T4^7F^ | P2487 | P2422 expressing type 7F capsule, streptomycin resistant | (3) |
| T4^14^ | P2488 | P2422 expressing type 14 capsule, streptomycin resistant | (3) |
| T4^23F^ | P2439 | P2422 expressing type 23 capsule, streptomycin resistant | (3) |
| T4^23F^ *cbpD::Janus* | P2647 | P2439 with Janus cassette replacing *cbpD*, kanamycin resistant | This study |
| T4^23F^ Δ*cbpD* | P2649 | P2647 with clean deletion of *cbpD*, streptomycin resistant | This study |
| T4^23F^ Δ*cbpD*Δ*cibABC* | P2653 | P2649 with Janus cassette replacing *cibABC* operon, kanamycin resistant | This study |
| T4^14^ *cbpD::Janus* | P2648 | P2488 with Janus cassette replacing *cbpD*, kanamycin resistant | This study |
| T4^14^ Δ*cbpD* | P2651 | P2648 with clean deletion of *cbpD*, streptomycin resistant | This study |
| T4^14^ Δ*cbpD*Δ*cibABC* | P2654 | P2651 with Janus cassette replacing *cibABC* operon, kanamycin resistant | This study |
| T4^23F^Δ*blpC* | P2678 | P2439 with Janus cassette replacing *blpC*, kanamycin resistant | This study |

References:

1. Zafar MA, Kono M, Wang Y, Zangari T, Weiser JN. 2016. Infant mouse model for the study of shedding and transmission during Streptococcus pneumoniae monoinfection. Infect Immun 84:2714-22.
2. Shen P, Lees J, Bee G, Brown S, Weiser J. 2019. Pneumococcal quorum sensing drives an asymmetric ownerintruder competitive strategy during carriage via the competence regulon. Nat Microbiol 4:198-208.
3. Zafar MA, Hamaguchi S, Zangari T, Cammer M, Weiser JN. 2017. Capsule type and amount affect shedding and transmission of Streptococcus pneumoniae. MBio 8:e00989-17.
