# Supplementary material for: Serotype-Dependent Effects on the Dynamics of Pneumococcal Colonization and Implications for Transmission
Source: mBio. 2022 Mar 15;13(2):e00158-22. doi: 10.1128/mbio.00158-22 (PMC9040870; doi:10.1128/mbio.00158-22)
Supplement: TABLE S2 [file mbio.00158-22-st002.docx]

**Table S2: Primers used in this study**

| **Purpose** | **Primer Name** | **Primer Number** | **Sequence** |
| --- | --- | --- | --- |
| Construction of *cbpD::Janus* and *cbpD* clean deletion strains | *cbpD* Upstream F | SDA12 | GAGTTGATTATCTTAGCAGC |
|  | *cbpD* Downstream R | SDA17 | ACTCAGATACCACAGTTG |
| Construction of *cibABC::Janus* strains | *cibABC* Upstream F | SDA10 | AATGCATACCAAGTCTGGTCTTG |
|  | *cibABC* Downstream R | SDA11 | TCTGGAATAGAGTGAGTCGTTCTAA |
| Construction of 𝚫*blpC* strain | *blpC* Upstream F | SDA01 | ATACCTTTATGAATTTTACTAAACAATCCCATG |
|  | *blpC* Downstream R | SDA09 | TCTCTTACTAAGATTAACTGGGCAATG |
